# Pandemic pushes polarisation: the Corona crisis and macroeconomic divergence in the Eurozone

**DOI:** 10.1007/s40812-020-00163-w

**Published:** 2020-07-10

**Authors:** Claudius Gräbner, Philipp Heimberger, Jakob Kapeller

**Affiliations:** 1 Institute for Socioeconomics, University of Duisburg, Essen, Germany; 2 Institute for the Comprehensive Analysis of the Economy (ICAE), Johannes Kepler University Linz, Linz, Austria; 3 ZOE, Institute for Future-Fit Economies, Bonn, Germany; 4 Vienna Institute for International Economic Studies (Wiiw), Vienna, Austria

**Keywords:** Polarisation, Coronavirus, Eurozone, E6, F4, O3

## Abstract

This paper discusses the uneven consequences of the macroeconomic fallout from the Coronavirus and related economic policy responses against the background of an analysis of longer-term macroeconomic divergence in the Eurozone. We show that the macroeconomic impact of the Corona crisis is estimated to be more severe in Southern Eurozone countries than in Northern Eurozone countries, which further reinforces the tendency of an increasing economic polarisation. This polarisation process can be traced back to existing differences in production structures and uneven vulnerabilities of the underlying growth models. As a consequence, any policy response to the Corona crisis that does not take the deeper problems of structural polarisation into account will suffer from limited impact in the medium to long run.

**Electronic supplementary material:**

The online version of this article (10.1007/s40812-020-00163-w) contains supplementary material, which is available to authorized users.

## Introduction

The Coronavirus and the resulting lockdowns and economic restrictions are severely testing the structural resilience of European economies. On the domestic level, the imposed restrictions tend to hit economically weaker households and firms harder, causing large-scale economic hardship that might fuel public resistance against economic restrictions based on public health concerns. Hence, social divisions may undermine the resilience of European societies in terms of public health on the level of domestic economies. Likewise, preliminary evidence on the European level suggests that economically weaker nations within the Eurozone are hit harder by the Corona crisis, which may have severe repercussions for the Eurozone as a whole. While this article focuses on the latter aspect—by asking how the Corona crisis may contribute to the amplification of economic polarisation within the Eurozone—a common observation worth spelling out in both the domestic as well as in the European context is that existing social divisions limit the collective resilience of societies in public health terms. In both contexts, weaker actors are not only hit harder, but have also fewer resources and leeway to cope with the immediate consequences of the crisis.

For the case of the Eurozone, the present article points out that because of the polarisation processes that started well *before* the Corona pandemic both the extent of existing vulnerabilities as well as the policy space to counter the crisis differ considerably across Eurozone member countries. As a consequence, the economic impacts are likely to be asymmetric and will, in the absence of coordinated policy responses, accelerate existing polarisation processes between an economically more well-off Northern and a struggling Southern Eurozone.^1^

**Fig. 1:**
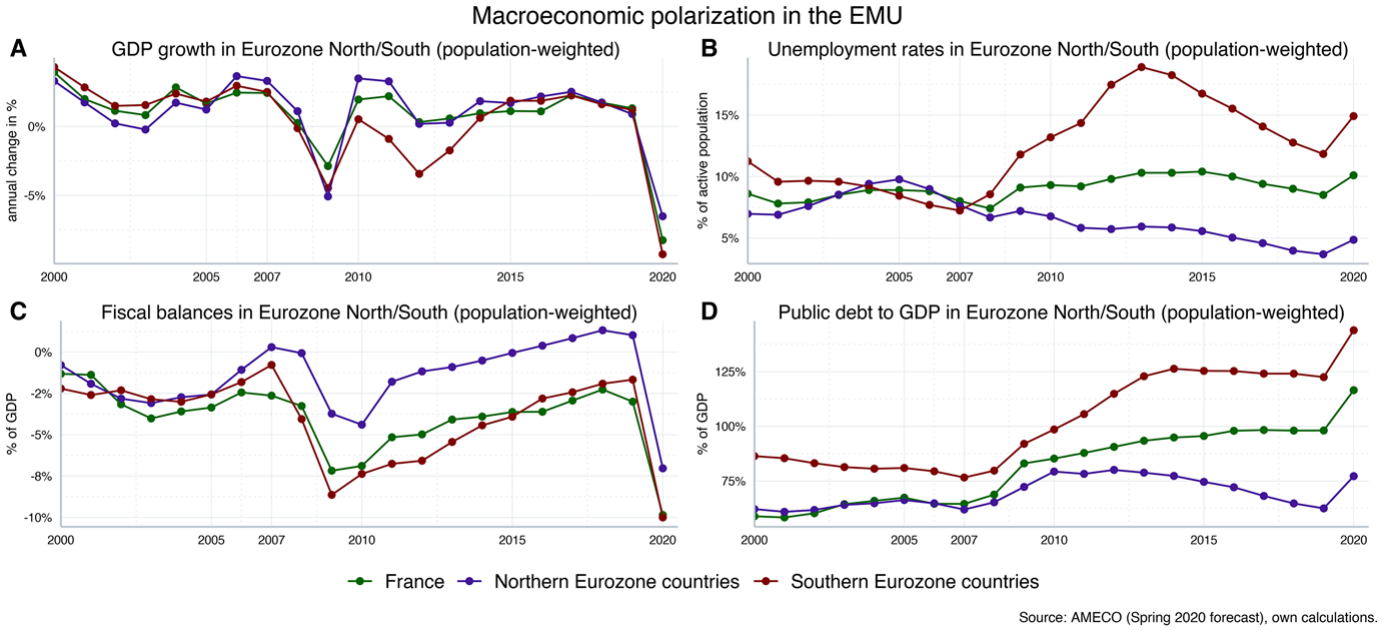
Macroeconomic polarisation in the Eurozone. Northern Eurozone (population-weighted average): Austria, Belgium, Finland, Germany, Netherlands. Southern Eurozone (population-weighted average): Greece, Italy, Portugal, Spain

The enormous challenge of economic recovery after the Corona health crisis will be most pressing in the Southern parts of the Eurozone, which consists of Greece, Italy, Portugal and Spain. In these Southern countries, the crisis is forecast to reduce GDP growth rates more than in the Northern Eurozone countries comprising Austria, Belgium, Finland, Germany and the Netherlands (see panel A of Fig. 1; since France often takes an intermediate position, it is reported separately). Furthermore, unemployment rates in Southern countries have not only reached much higher levels in pre-Corona times as compared to the Northern Eurozone, they also seem to be more strongly affected by the advent of the Corona crisis: according to the most recent macroeconomic forecasts^2^ Southern countries will suffer a relatively more pronounced increase in unemployment due to the economic downturn, aggravating the already existing differences in the Eurozone (see panel B of Fig. 1). Thus, recovery needs differ considerable across regions in Northern and Southern parts of the Eurozone (European Commission 2020a).

**Fig. 2:**
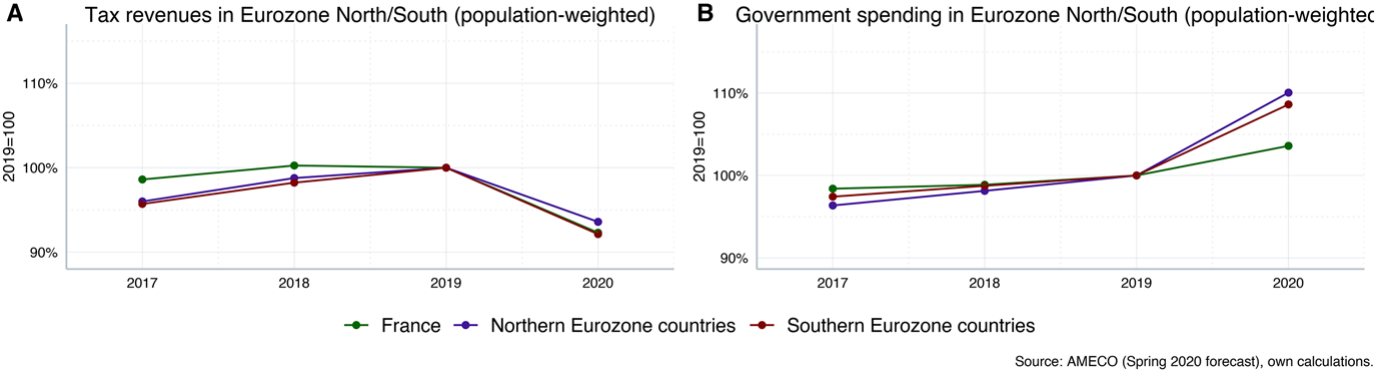
Tax revenues and government spending in the Eurozone (inflation-adjusted). Northern Eurozone (population-weighted average): Austria, Belgium, Finland, Germany, Netherlands. Southern Eurozone (population-weighted average): Greece, Italy, Portugal, Spain

At the same time, Southern Eurozone countries—above all Italy and Greece—have entered into the Corona crisis with high levels of public debt. Recent forecasts suggest that increases in fiscal deficits in the Southern periphery will be particularly severe due to increasing government spending and decreasing tax revenues, and public-debt-to-GDP ratios—which are quantitatively affected by both increasing fiscal deficits as well as decreasing GDP—will rise strongly (see panels C and D of Fig. 1). Furthermore, government revenues are expected to decline more in Southern Eurozone countries against the background of bigger losses in economic activity than in Northern Eurozone countries. At the same time, government spending is forecast to increase more in the Northern Eurozone countries, reflecting a stronger response by automatic stabilizers as well as bigger discretionary efforts to counteract the Corona crisis (see Fig. 2).

**Fig. 3:**
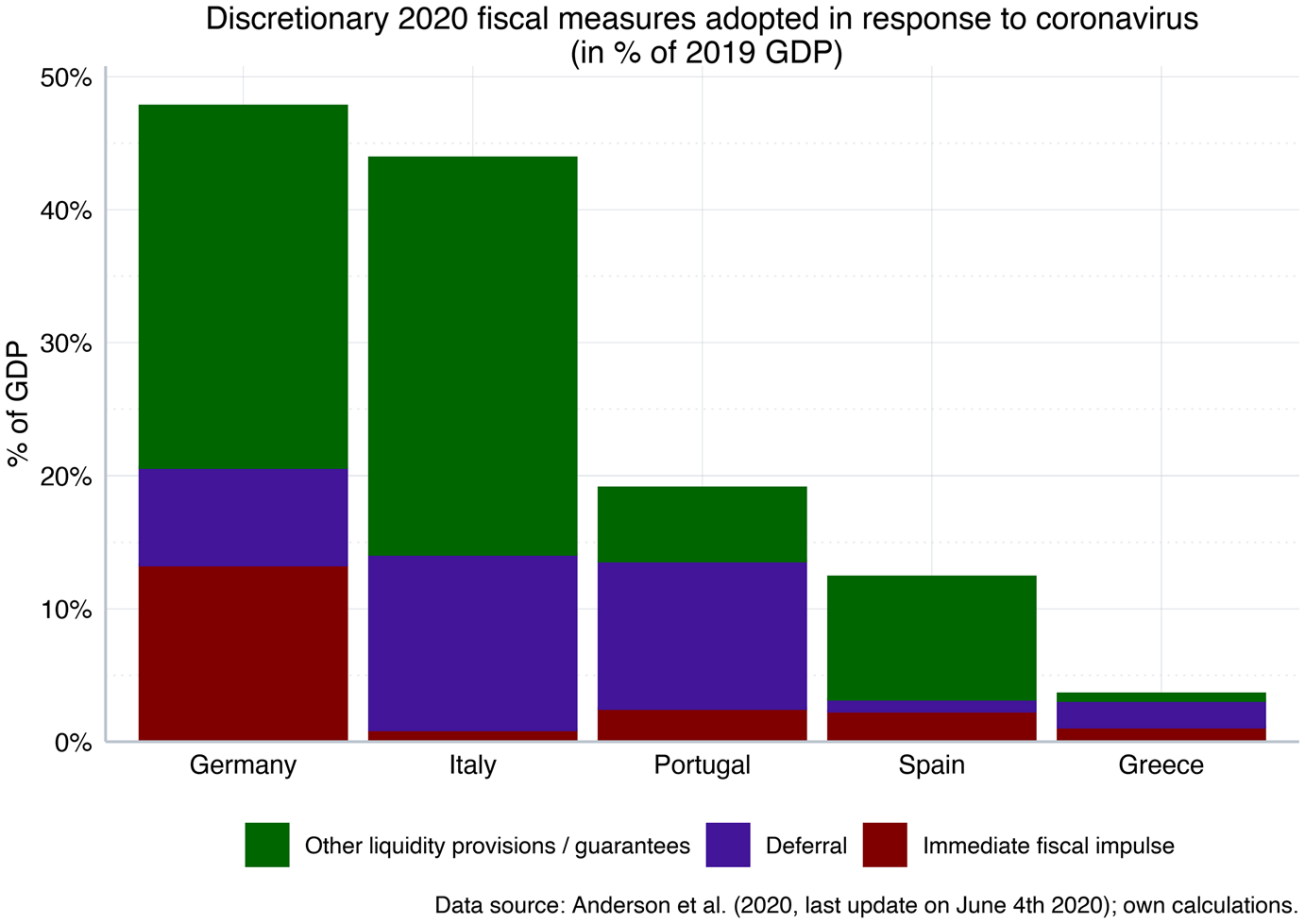
The fiscal response to the economic fallout from coronavirus: Germany vs. Southern Eurozone countries. Immediate fiscal impulse: additional government spending (such as medical resources, short-term work, subsidies for companies, public investment). Deferral: tax and social security contributions deferral. Other liquidity provisions and guarantees: Export guarantees, liquidity assistance, credit lines through national development banks

Against this background, there is a risk that—under current institutional conditions—Italy and other Southern Eurozone countries will be able to finance only the most urgent measures, while Northern Eurozone countries with a better starting position—especially Germany, Austria or the Netherlands—have more fiscal space to support a rapid recovery once the economy is jump-started. In this view the main constraints that prohibit a quick(er) recovery of Southern countries arise from the current institutional arrangements under which fiscal space is typically correlated with macroeconomic performance (Heimberger and Kapeller 2017).^3^ Available data already point to such asymmetric fiscal responses at the national level: in particular, the immediate increase in fiscal spending in Germany (in the form of additional government spending on medical equipment, short-time work, subsidies for small and medium-sized enterprises etc.) amounts to more than 10% of economic output in 2020, compared with only 0.9% for Italy, 1.1% in Spain, 2.5% in Portugal and 1.1% in Greece. But also the indirect fiscal response—the deferral of taxes and social security contributions as well as other liquidity provisions and loan guarantees—lags behind Germany in all Southern countries except Italy (see Fig. 3, based on Anderson et al. 2020). While the numbers shown in Fig. 3 are influenced by a series of qualitatively different factors—including the impact of the pandemic and the state of the public health systems—they also reflect differences in fiscal space across countries, especially in the context of direct spending undertaken by governments. This observation suggests that existing differentials in economic performance are indeed aggravated through the Corona pandemic and that the competitiveness as well as the standard of living in the Southern countries is likely to deteriorate further relative to other parts of the Eurozone.

This article discusses these uneven macroeconomic consequences and economic policy responses against the background of an analysis of longer-term macroeconomic divergence in the Eurozone. Previous research has shown that the underlying processes are path-dependent and relate not only to the divergence of major macroeconomic indicators, but also to the polarisation of production structures between Eurozone member countries and the associated development of divergent export-led and private-debt-led growth models (Simonazzi 2013; Botta 2014; Storm and Naastepad 2015; Celi et al. 2018; Gräbner et al. 2020b). The present paper also highlights that increased macroeconomic polarisation in pre-Corona years has fuelled political polarisation, which has become visible in recent Corona policy debates concerning the appropriate response to the macroeconomic consequences of the COVID-19 pandemic: countries such as Italy and Spain have immediately pushed for a stronger common European fiscal response, only to find their more ambitious proposals about European burden-sharing of the crisis costs turned down by Northern Eurozone countries. A more nuanced discussion about the potential for a pan-European recovery initiative only started with a considerably time lag, promoted by a change in the political stance of the German government. It will be argued below, however, that—in the absence of coordinated policy interventions—the process of economic divergence occurring in the Eurozone must be expected to accelerate further after the lockdown. Such a process would put the Eurozone as a whole at risk. With an eye to preventing the common currency area from falling part, we will discuss some elements of coordinated fiscal and industrial policy action that could contribute to countering economic polarisation in the context of the Corona crisis. Such policies could also be designed in a way that is consistent with a longer-term orientation towards achieving social and environmental sustainability.

## Structural polarisation and growth models before and after Corona

This section analyses structural polarisation and macroeconomic divergence in the Eurozone in conjunction with different growth models. It begins with an analysis of structural polarisation processes in pre-Corona years and continues with an analysis of the impact of the pandemic.

### Structural polarisation before Corona

**Fig. 4:**
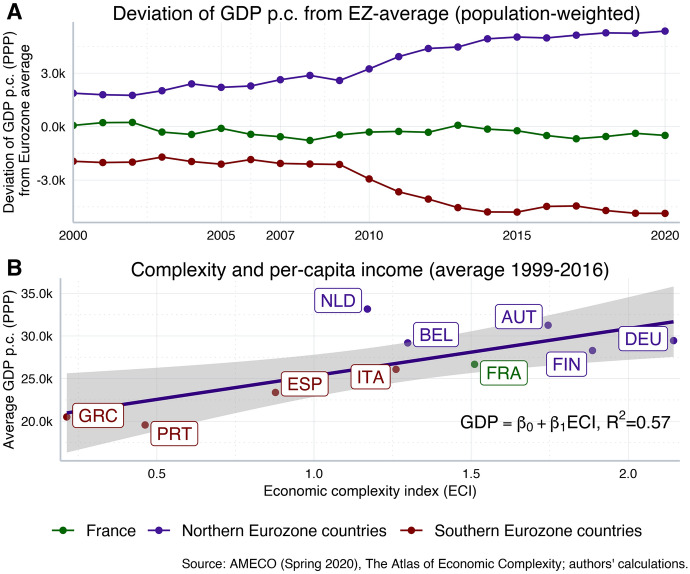
GDP per capita and economic complexity. Northern Eurozone (population-weighted average): Austria, Belgium, Finland, Germany, Netherlands. Southern Eurozone (population-weighted average): Greece, Italy, Portugal, Spain

The gap in per capita incomes between Northern and Southern Eurozone countries has widened considerably since the birth of the Euro about twenty years ago (see Fig. 4a). Particularly the 10 years before the Corona crisis have been characterised by a persistent divergence in terms of living standards of large parts of the Eurozone, which can be traced back to the co-existence of distinct *growth models* within the EMU: Southern European countries mainly followed *debt-led* growth models, which came with increasing private-sector indebtedness, linked to the accumulation of current account deficits (e.g. Storm and Naastepad 2016). At the same time, Northern countries mainly followed *export-led* growth models, which came with current account surpluses and a stronger reliance on foreign trade. When the financial crisis and the subsequent Euro crisis hit, the fragility of these imbalances built up in pre-crisis times laid bare the underlying reality of macroeconomic divergence (Gräbner et al. 2020b).

Essential factors for explaining the long-term divergence of Eurozone countries are to be found in the unequal regulatory conditions in the context of the European ‘race for the best location’ (for example, in the areas of the labour market, tax and corporate law, or financial market regulation, see Kapeller et al. 2019) as well as in the different technological capabilities across EU countries (Gräbner et al. 2019, 2020b). Technological capabilities serve as an important driver of long-term economic development, and there exists a strong positive relationship between the level of economic complexity (used as a proxy for technological capabilities; see Hidalgo and Hausmann 2009) and GDP per capita levels (see Fig. 4b). The problem in the decades running up to the Corona crisis has been that the accumulation of these technological capabilities is a highly path-dependent process: in the absence of coordinated policy measures, existing differentials in technological capabilities will be self-reinforcing over time, particularly within the Eurozone, where traditional compensation mechanisms for individual member countries are either not available (currency devaluations) or severely restricted (fiscal and monetary policy) due to existing institutional arrangements.

Technological capabilities are also relevant when it comes to explaining the emergence of the unsustainable co-existence of export-driven and debt-driven growth models in the EMU: countries in the North were better equipped to follow an export-led growth model precisely because their economies have accumulated a sufficient amount of the technological capabilities necessary to compete successfully on international markets, where technological sophistication is more important than price competitiveness (Storm and Naastepad 2015; Dosi et al. 2015; Gräbner et al. 2020b). Furthermore, Northern euro countries—most of all, Germany—were able to strive over recent decades not despite, but also because of the rise of Asian economies such as China: for firms that have focused on the production of technologically complex products, the rise of Asian countries came with additional export opportunities to Asian partners, who were keen to acquire more complex products and capital goods. Technological sophistication of firms in Northern Eurozone countries represented a unique competitive advantage in the global economy that remains relevant until today.

The emergence of these different growth models also had a feedback effect on the further development of production structures: while Germany and other Northern countries have expanded their cumulative advantage in high-tech manufacturing over the past 2 decades, Southern European countries have increasingly been locked into lower-tech and non-tradable activities (e.g. Simonazzi 2013; Botta 2014). As a consequence, Northern firms often do not directly compete with Spanish, Portuguese, Greek or even most Italian firms; instead, they are price-setters due to their strong market standing, which is generated by a high degree of technological sophistication. In contrast, firms located on the Southern periphery (e.g. Greece and Portugal) are more often confined to the role of price-takers, as they compete with low-cost Asian producers (Straca 2013). As a consequence, they were much less able to base their competitiveness on technological capabilities, while competing in terms of low wages (or reduced environmental or labour protections) would also be economically infeasible and politically destructive given the current levels of wages and regulations in Europe.

In summary, most European firms with a strong technological position typically operate from their home base in Northern countries, such as Germany and Austria. Despite important exceptions, particularly in the industrial North of Italy and Spain, many firms in the Southern Eurozone are relative technological laggards and the overall international competitiveness of Southern economies has deteriorated. Due to the cumulative nature of the underlying processes, existing differences in technological capabilities are to be seen as both a driving factor as well as a major outcome of long-term divergence within the Eurozone (Gräbner et al. 2020b), which is reflected in increasing macroeconomic polarisation as captured by Fig. 4.

### The asymmetric impact of the Corona Crisis

**Fig. 5:**
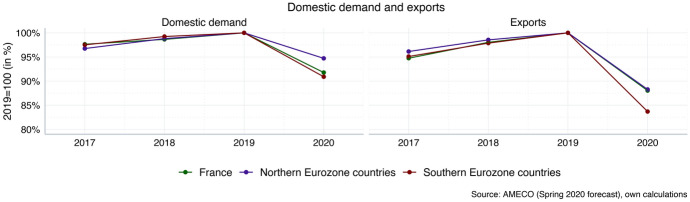
Domestic demand and exports after Corona (inflation-adjusted). Northern Eurozone (population-weighted average): Austria, Belgium, Finland, Germany, Netherlands. Southern Eurozone (population-weighted average): Greece, Italy, Portugal, Spain

The macroeconomic impact of the Corona crisis on Northern and Southern Eurozone countries will be asymmetric due to existing differences in production structures and because of the vulnerabilities of the different growth models described above. The most recent macroeconomic forecasts suggest that domestic demand will take a bigger hit in the Southern Eurozone than in Northern Eurozone countries (see Fig. 5). Given their relatively strong reliance on domestic demand as compared to exports, this implies a particular challenge for these economies. However, Germany and other Northern Eurozone countries will also not be able to rely on export-driven growth to the same extent as in the years 2010–2019, because China and other emerging Asian economies also suffer greater economic losses, which decreases their demand for imports. Moreover, the global economy as a whole has been hit hard by the repercussions of the coronavirus (International Monetary Fund 2020), and the partial disruption of global value chains will make an export-based recovery strategy more difficult to implement in the medium term as well. However, the data in Fig. 5 suggest that the overall challenge is considerably more difficult for countries in the South, as domestic demand takes a bigger hit and exports decline more strongly.

**Fig. 6:**
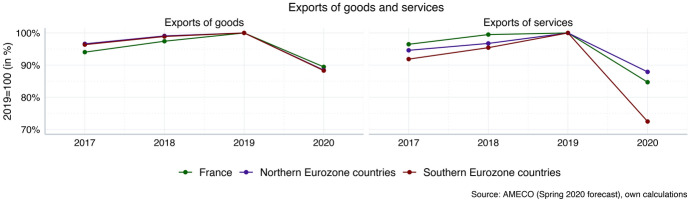
Exports of goods and services after Corona (inflation-adjusted). Northern Eurozone (population-weighted average): Austria, Belgium, Finland, Germany, Netherlands. Southern Eurozone (population-weighted average): Greece, Italy, Portugal, Spain

Figure 6 provides a breakdown of declining exports in terms of exports of goods and exports of services, respectively. Thereby it points to yet another way in which structural polarisation patterns from the pre-Corona years provide the conditions for an asymmetric effect of the Corona shock: while exports of goods are set to decline to a similar extent in Northern and Southern countries, the much stronger drop in exports of services in the South exposes another vulnerability. The prospects for booming global markets for export goods have deteriorated, but the prospects for exports of services—in particular, concerning the tourism-related sectors—may be even gloomier because of shifting preferences for tourist destinations and the restrictions imposed on international travel. Prolonged restrictions will have disproportionately strong negative effects on the regions in Southern Europe, which clearly raises the prospect of accelerating macroeconomic divergence (Odendahl and Springford 2020).^4^ Moreover, while at least some of the goods that have been produced for export but have not been sold yet can be put in storage and still represent an increase in value-added in the future, services that have not been demanded in the present tend to be lost forever. This suggests that the recovery process for Southern countries will be more difficult than in Northern countries.

## Policy options and conclusions

The analysis above suggests that—in the absence of adequate coordinated European policy interventions—the Corona crisis will contribute to a further deepening of macroeconomic divergence and structural polarisation between Northern and Southern Eurozone countries. As economic polarisation fuels political polarisation, this process puts the survival of the Eurozone as a whole at risk. In what follows, we briefly evaluate European economic policy initiatives in response to the Corona shock. In this context, we do not only inspect short-run issues but also explore whether and to what extent current policy measures are suitable for addressing the root causes of economic polarisation within the Eurozone by drawing on a holistic analysis of policy-challenges induced or aggravated by this polarisation—as developed by Kapeller et al. (2019) and summarized in Table 1 below.

**Table 1:** The role of Corona-related policy practices/suggestions in combatting economic polarisation in the Eurozone based on Kapeller et al. (2019)

Policy area	Policy suggestions	Relation to actual policies induced by the Corona crisis
Strengthen European Values	Reduce competition and increase cooperation between EU member states	Not yet, but potentially lurking in the background of current discussions
	Base trade policies on human rights considerations (“civilised trade”)	None
	Achieve greater equality in earned incomes	Partly visible efforts on domestic level (increased unemployment benefits, suspension of dividends)
Thinking the Eurozone through	Common monetary and fiscal policy	Creation of new credit lines in ESM, discussion of joint financing procedures
	Reorient monetary policy towards greater prosperity	Failure to do so significantly limits instruments and impact of the ECB
	Reregulating financial markets	None
Ending the European *race for the best location*	Promoting sustainable industries and regional development by industrial policies	Unspecific references to green and digital transitions and building “European industrial champions”
	Coordinate tax policy and combat tax avoidance	None
	Avoiding permanent current account imbalances	None
Rethinking the Economy	Progress vs. GDP: new concepts of prosperity	None
	Actively govern future socio-economic challenges (climate, ecology, automatisation etc.)	Unspecific references to green and digital transitions
	Explore the trade-off between welfare in time and welfare in goods to foster a sustainable transformation	None

The first immediate European policy responses to the Coronavirus came from the ECB, which started to buy up government bonds on a large scale: the PEPP programme (“Pandemic Emergency Purchase Programme”) was put in place to prevent rising interest rate spreads and to “ensure the smooth transmission of monetary policy to the economy” (Lane 2020). It serves to ensure that governments in the Southern Eurozone can continue to refinance at low interest rates during the Corona crisis. However, as indicated by our analysis above, Southern Eurozone countries such as Italy and Spain will not be able to get the economy back on track after the Corona lockdown with the simple provision of cheap credit. The EU’s fiscal rules have been temporarily suspended, but Southern countries are still suffering from legacy debt and problems related to structural polarisation, which will become even more apparent when the fiscal rules suspension is lifted to further reduce their fiscal space. In sum, the ECB is, as in past crises, again acting as a lender of last resort to hold the Eurozone together. However, existing institutional arrangements still pose constraints on the actions of the ECB. Therefore, the Corona crisis opens up a window for discussing a modified mandate for the ECB, which widens it from a primary focus on price stability to also include a commitment to maximum employment and environmental sustainability. However, such discussions currently remain subordinated to the question about the short to medium run effects of the German Constitutional Court’s recent ruling that the ECB has failed to adequately justify its Quantitative Easing Program (Tooze 2020).

In addition to the ECB’s actions, European leaders have decided on a package of loan assistance amounting to 540 billion euros. A new ESM credit line has been established (up to 240 billion euros), which—although only subject to minor conditionality—will be limited to covering “direct and indirect” health costs. However, government spending on healthcare costs will not play a major role in the bigger picture of the costs of the crisis. Furthermore, it is doubtful whether a country like Italy would ever use such a credit line, because the ESM is seen to be “politically toxic” due to memories going back to the Euro Crisis. In addition, there is a new EU programme to grant member states cheap loans without conditions to support short-time work, which is called SURE (Support to Mitigate Unemployment Risks in an Emergency). This will enable the EU to borrow on financial markets and to pass on the funds to the member states. Furthermore, the package consists of loan guarantees from the European Investment Bank for companies.

Even if European loans came with cheap conditions and light or no conditionality (where especially the point concerning light conditionality remains doubtful), they will nevertheless further increase public debt levels in Southern euro countries hit hard by the pandemic. Much of the discussion concerning longer-term questions of European burden sharing has, therefore, revolved around the establishment of a so-called “recovery fund” and the possibility of grants. However, even if we assume that a sizeable recovery fund including a component of grants for the regions and sectors hit hardest by the pandemic is implemented over the horizon of the next couple of years, such a short-run policy instrument would still prove insufficient if the goal is to reverse the underlying path-dependent process of polarisation between North and South. Without addressing the deeper problems of structural polarisation analysed in this paper, any policy response to the Corona crisis will suffer from limited impact in the long run. To this end, Table 1 lists the policy suggestions for addressing polarisation as discussed in Kapeller et al. (2019) and compares them with actual Corona-related policy measures and discussions.

Comparing the policy measures currently undertaken or planned with the long-term challenges arising from economic polarisation in the Eurozone reveals that the current policy focus is mostly on short-run measures to keep the economy going and/or to jumpstart economic activities after lockdowns and other restrictions have been sufficiently released. More medium- or long-term considerations currently play a subordinate role, although crises like the current one always carry the potential to induce greater reflexion among policy-makers and social elites of all kind. What seems most urgent given the current focus on organizing the means for significant public investment in economic recovery is to tie these funds to important medium-term concerns, such as the reorganisation of global value chains, the expansion of industrial policies to combat technological divergence on the regional and national level or, probably most importantly, recasting European economies in a way that is compatible with planetary boundaries in the context of climate change. And indeed, such links between pressing immediate demands and medium-term strategic challenges also partially coin the viewpoint of the European Commission (e.g. European Commission 2020b), which has tried to connect the Corona-response with its longer-term vision of a European Green Deal. However, these connections remain relatively unspecific and underdeveloped as they do neither indicate specific strategies for reaping the alleged synergies, nor have they led to a commitment towards increased funding of the European Green Deal. Such a commitment, however, seems highly necessary given that the current funding lags behind the investment requirements for achieving relevant goals concerning the reduction of CO_2_ emissions (Wildauer et al. 2020). However, if such links between the Corona-response and the European Green Deal could be established and further developed, the hopefully occurring recovery from the Corona-crisis could have positive spill-over effects that would be of great merit for confronting the socio-economic challenges around the ‘Corona-corner’.

## Electronic supplementary material

Below is the link to the electronic supplementary material.

Supplementary file1 (PDF 312 kb)

## Footnotes

1 Our focus in this paper is on Northern and Southern countries. Gräbner et al. (2019) suggest that when taking into account all EU countries a taxonomy of four groups is more adequate. The proposed taxonomy also accounts for countries serving as financial hubs (where the financial sector plays an outstanding role) and Eastern European countries that partially experience catching-up dynamics. All figures in the present paper are also provided with this more complete country grouping in the appendix. While this modification does not affect the core message of our article, it indicates that the polarisation problem in Europe goes beyond a simple North-South division.

2 All the macroeconomic forecast data used in this paper are characterized by higher uncertainty than usual, because the COVID-19 pandemic also pushes economic forecasters into unchartered waters, and they may turn out to be too optimistic.

3 The EU’s fiscal rules have been temporarily suspended. However, Southern Eurozone countries record larger public legacy debt than Northern Eurozone countries, which will become an even bigger constraint for the existing policy space once the suspension of the fiscal rules is lifted.

4 Fiscal consolidation efforts in pre-Corona times implied significant deteriorations of health care systems in Southern Eurozone countries such as Italy. This may partly also explain that it is more difficult for Southern Eurozone countries to deal with the health crisis that results from the COVID-19 pandemic (e.g. Prante et al. 2020).
