# Supplementary material for: Pandemic pushes polarisation: the Corona crisis and macroeconomic divergence in the Eurozone
Source: J. Ind. Bus. Econ. 2020 Jul 10;47(3):425–38. doi: 10.1007/s40812-020-00163-w (PMC7347665; doi:10.1007/s40812-020-00163-w)
Supplement: Supplementary file 1 — Supplementary file1 (PDF 312 kb) [file 40812_2020_163_MOESM1_ESM.pdf]

Pandemic pushes polarisation:

The Corona crisis and macroeconomic divergence in the Eurozone*

Supplementary material

Claudius Gräbner[a,b,c] Philipp Heimberger[b,d] Jakob Kapeller[a,b]

[a] *Institute for Socioeconomics, University of Duisburg-Essen, Germany*
[b] *Institute for the Comprehensive Analysis of the Economy (ICAE), Johannes Kepler University Linz, Austria*
[c] *ZOE. Institute for Future-Fit Economies, Bonn, Germany*
[d] *Vienna Institute for International Economic Studies (wiiw), Vienna, Austria*

Emails for correspondence:
claudius@claudius-graebner.com (CG), heimberger@wiiw.ac.at (PH), jakob.kapeller@uni-due.de (JK)

Content

Here we provide all the figures of the main paper using data for the EU27 countries by using the country classification as introduced in Gräbner et al. (2019). While this modification does not affect the core message of our article, it indicates that the polarisation problem in Europe goes beyond a simple North-South division. Information about the country selection and country groups used in the main paper as well as in the supplementary material are provided in Table 1 and Table 2.

**Country group**	**Countries**
**Northern Eurozone countries**	Austria, Belgium, Finland, Germany and the Netherlands
**Southern Eurozone countries**	Greece, Italy, Portugal and Spain

Table 1 The country classification as used in the main text. Since France often takes an intermediate position it is reported separately.

* Supported by funds of the Oesterreichische Nationalbank (Austrian Central Bank, Anniversary Fund, project number: 18144) and the Austrian Science Fund (FWF, grant number ZK 60-G27). Data and code to replicate the findings of the paper are available via Github (graebnerc/structural-corona-crisis) and published as Gräbner et al. (2020).

Country group	Countries
**Core countries**	Austria, Belgium, Denmark, Finland, Germany, and Sweden
**Periphery countries**	Cyprus, France, Greece, Italy, Portugal, and Spain
**Catch-up countries**	Bulgaria, Croatia, Czechia, Estonia, Hungary, Latvia, Lithuania, Poland, Romania, Slovakia, and Slovenia
**Financial hubs**	Ireland, Luxembourg, Malta, and the Netherlands

Table 2 The country classification as used in the supplementary material. This classification has been derived and further explained in Gräbner et al. (2019).

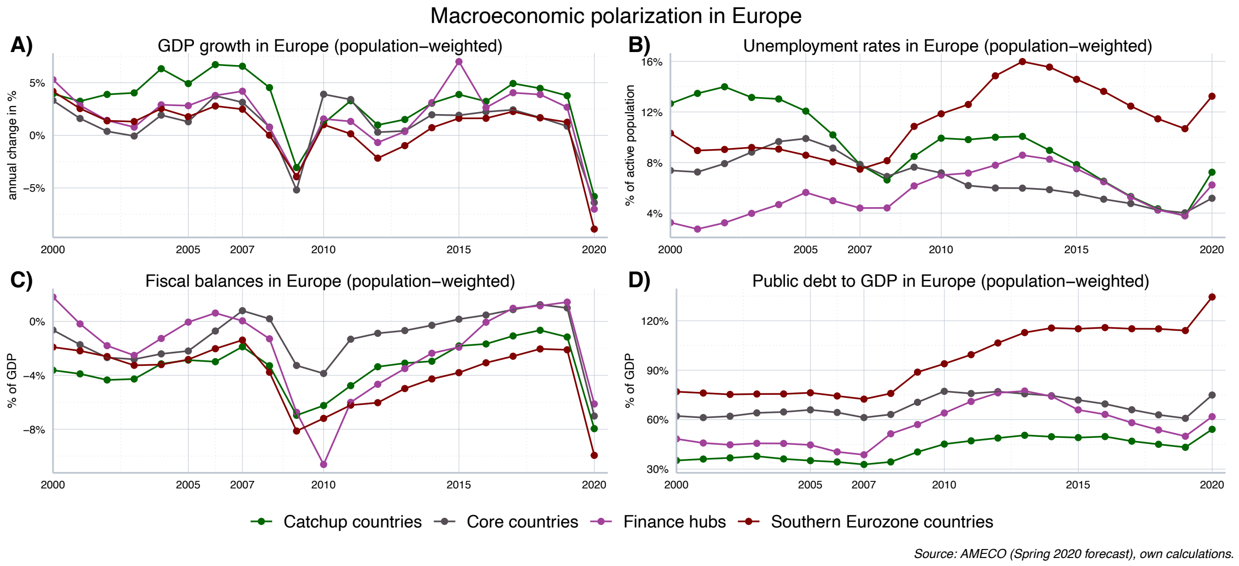

Figure 1 Macroeconomic polarization in Europe. Corresponds to figure 1 in the main paper.

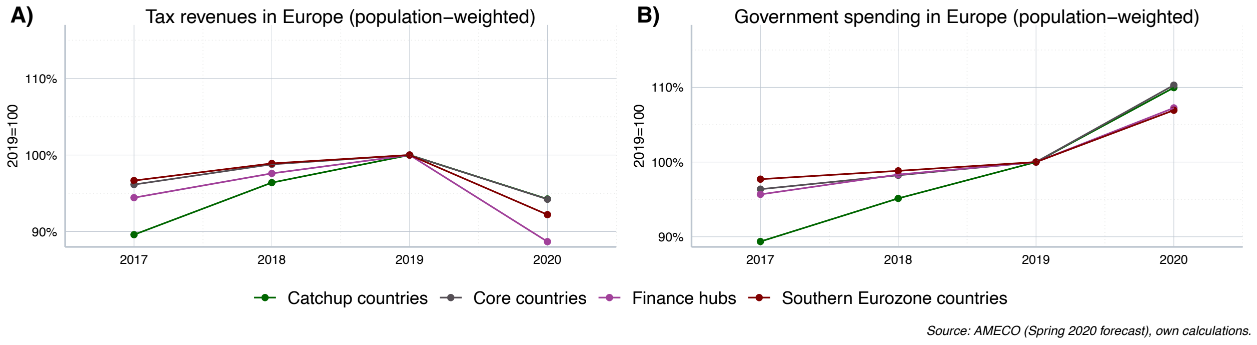

Figure 2 Tax revenues and government spending. Corresponds to figure 2 in the main paper.

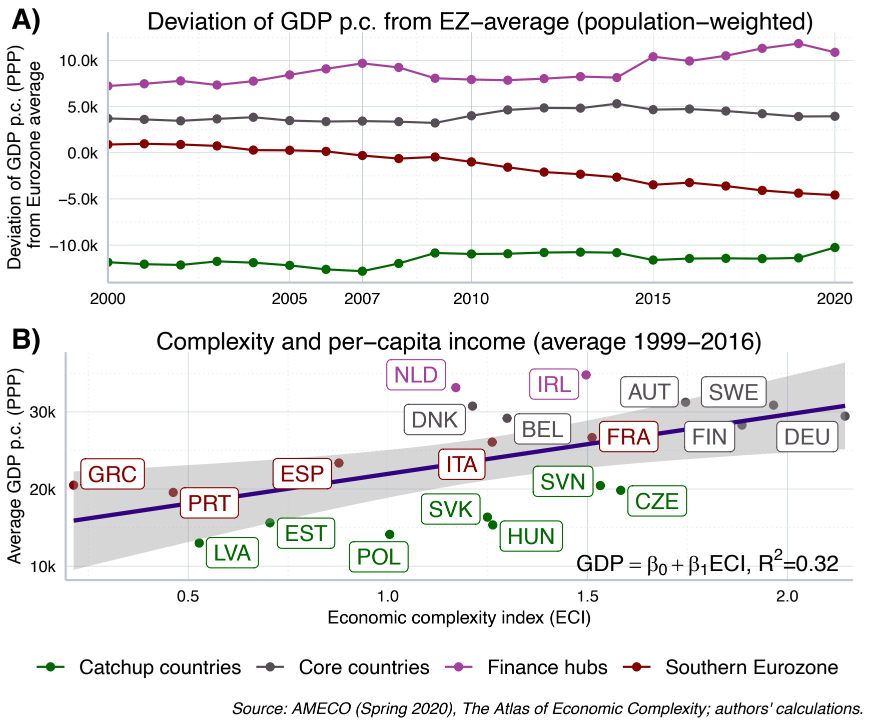

Figure 3 GDP per capita and economic complexity. Corresponds to figure 4 in the main paper.

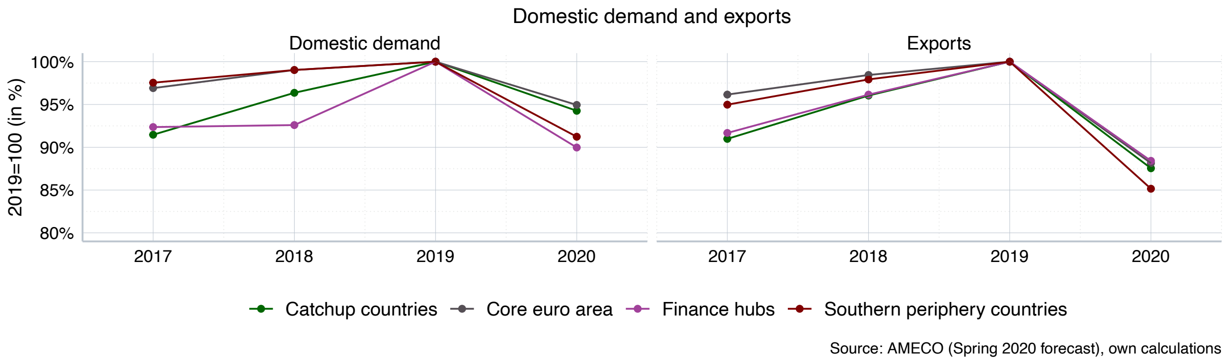

Figure 4 Domestic demand and exports after Corona. Corresponds to figure 5 in the main paper.

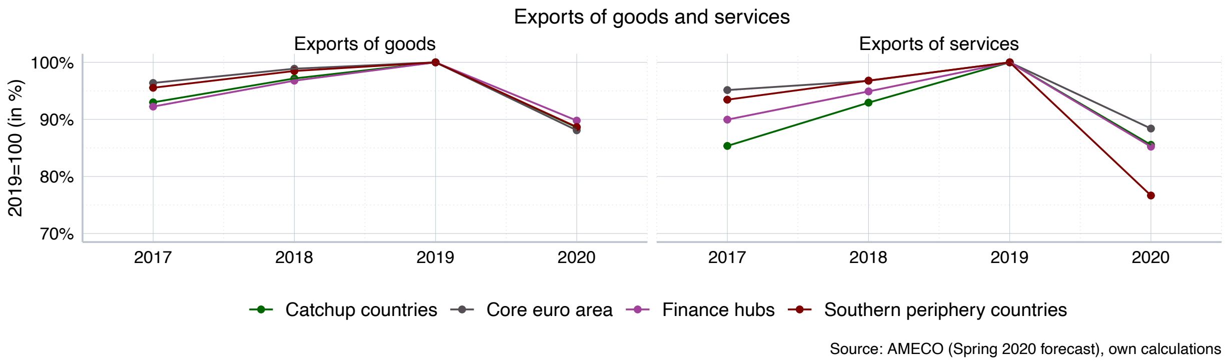

Figure 5 Exports of goods and services after Corona. Corresponds to figure 6 in the main paper.

References

Gräbner, C.; Heimberger, P.; Kapeller, J.; Schütz. B. 2019. Structural change in times of increasing openness: assessing path dependency in European integration, *Journal of Evolutionary Economics, forthcoming,* doi: https://doi.org/10.1007/s00191-019-00639-6

Gräbner, C., Heimberger, P., Kapeller, J., Schütz, B. (2020): Pandemic pushes polarisation: The Corona crisis and macroeconomic divergence in the Eurozone, *Harvard Dataverse*, doi: 10.7910/DVN/G2VMKH
